# Importance of incorporating spatial and temporal variability of biomass yield and quality in bioenergy supply chain

**DOI:** 10.1038/s41598-023-28671-4

**Published:** 2023-04-26

**Authors:** Mohammad S. Roni, Yingqian Lin, Damon S. Hartley, David N. Thompson, Amber N. Hoover, Rachel M. Emerson

**Affiliations:** 1 System Science & Engineering Department, Idaho National Laboratory, Idaho Falls, ID USA; 2 Biomass Characterization Department, Idaho National Laboratory, Idaho Falls, ID USA

**Keywords:** Environmental sciences, Environmental impact

## Abstract

Biofuels made from biomass and waste residues will largely contribute to United States’ 2050 decarbonization goal in the aviation sector. While cellulosic biofuels have the potential fuel performance equivalent to petroleum-based jet fuel, the biofuel industry needs to overcome the supply chain barrier caused by temporal and spatial variability of biomass yield and quality. This study highlights the importance of incorporating spatial and temporal variability during biomass supply chain planning via optimization modeling that incorporates 10 years of drought index data, a primary factor contributing to yield and quality variability. The results imply that the cost of delivering biomass to biorefinery may be significantly underestimated if the multi-year temporal and spatial variation in biomass yield and quality is not captured. For long term sustainable biorefinery operations, the industry should optimize supply chain strategy by studying the variability of yield and quality of biomass in their supply sheds.

## Introduction

As the world’s climate changes, an impact to weather patterns and the frequency and severity of precipitation events are expected. Within biomass supply chains, the potential effects of climate change may result in impacts to the quantity and spatial distribution of the materials within the supply region. While the increased CO${_2}$ will likely enhance the yield, drought and heat stresses will likely have negative impacts on the yield^1^. Additionally, biomass chemical composition (e.g. cellulose, hemicellulose, and lignin) are likely to exhibit higher variablility due to responses to the stressors^2^. As the effects of climate change become more frequent and severe, it is important that the supply systems are designed to be resilient to these impacts.

Lignocellulosic biomass such as—agricultural residues, woody biomass, and dedicated energy crops—are known as promising renewable resources for cellulosic ethanol production and are considered a key solution for increasing energy security and reducing dependence on fossil fuels. Therefore, considerable efforts have been made over the last few decades to develop efficient and cost-effective technologies and to establish cellulosic ethanol on a commercial scale^3^. Despite considerable investment cellulosic biorefinery is not springing up across the United States. Among many reasons, variability in biomass in addition to its underlying economic impact has prevented attracting investment in the cellulosic biorefining industry. Increasing the resilience of the biomass supply chain allows biorefineries to control their operational cost and quality envelope facing uncertainties caused by climatic conditions.

It has been concluded in previous studies that key uncertainties in biofuel supply chain include seasonal variability in biomass supply, pre-treatment uncertainties, production and yield uncertainties^4^. Many approaches and models have been developed that focus on uncertainty modeling and stochastic optimization of biofuel supply chains. For example^5^, has applied the Bayesian network theory to simulate the probability of random risks such as floods and earthquakes, and^6^ has applied Monte Carlo simulation to generate optimization scenarios with different biomass demand and prices. Multi-stage or multi-period stochastic programming is commonly used to capture seasonal uncertainties and enable supply chain planning^4,7^. For instance^8^, developed a 2-stage chance-constraint optimization model to ensure that the used municipal solid waste quantity is above certain threshold while minimizing total supply chain cost^9^. developed a multi-stage optimization model that considered both spatial and temporal variability of biomass demand and supply in 10 years and illustrated the impact of long-term supply chain strategic planning. However, most of these previous studies have mainly focused on only biomass supply in terms of quantity, not quality, which may largely impact the actual cost of biomass pre-processing and conversion yield.

### Yield variability

Variability in feedstock yield is often considered as a major source of risk for biorefinery investors, as it can directly affect biomass availability and feedstock costs for biofuel production and biorefinery operations^10^. A higher and more stable biomass supply can potentially improve the economic performance of biorefining with an expected annual return on investment (ROI) of up to 50%^11^. Since large-scale biofuel production requires large amounts of feedstock, it is critical to evaluate and consider the biomass yield variability in the biomass supply chain design. To mitigate the impacts of supply risk on biofuel production, research has been done to analyze different supply chain configurations, such as centralized and distributed biomass processing depots. Study indicates that switching to a distributed supply system can reduce the operational risk of a biorefinery by 17.5%^12^. Several studies have also been conducted to select the optimal plant size and location for biorefineries or biomass processing depots^13–15^; however, to our knowledge, none of these studies considered long-term spatial and temporal yield variability in their decision making, which could lead to non-robust solutions to manage biomass supply risk.

Advanced supply systems like depots can be used to minimize biomass supply risk and support higher and less variable crop yields when weather events like drought occur^16^. Over 60% of crop yield variability is attributable to weather variability^17^; therefore, in biomass supply chains, it is extremely important to consider how crop yield variability is affected by climate change and extreme weather events. Yield variability can be due to many spatial and temporal factors, such as weather, drought events^2,18^, mean air temperature^18^, growing degree days, soil temperature^19^, soil characteristics, landscape factors, and field management practices and history. Water stress caused by low precipitation and drought can reduce crop yields and even alter plant cells and overall chemical composition. The combination of low humidity and soil water deficit during drought contributes to a myriad of short-term and long-term impacts as plants try to avoid dehydration and oxidative stress. In the short-term, signal recognition and gene responses in the foliage and roots occur as plants close their stomata, decrease carbon assimilation, inhibit growth, and experience xylem hydraulic and osmotic adjustments^20^. Long-term, plants will promote root growth while inhibiting shoot growth and maintaining osmotic adjustments and turgor pressure^20^. In a meta-analysis conducted by^21^, it was highlighted that drought and heat stress during crop growth can reduce crop yields by up to 48% and harvest index by 28%, shorten the crop life cycles (e.g., time to reach crop maturity), and change crop chemical compositions, such as reducing starch up to 60%. In addition, for $\textrm{C4}$ crops such as corn, miscanthus, and switchgrass, yield losses due to drought cannot be alleviated by increasing ${\textrm{CO}}_{2}$ levels as expected^18^.

**Figure 1:**
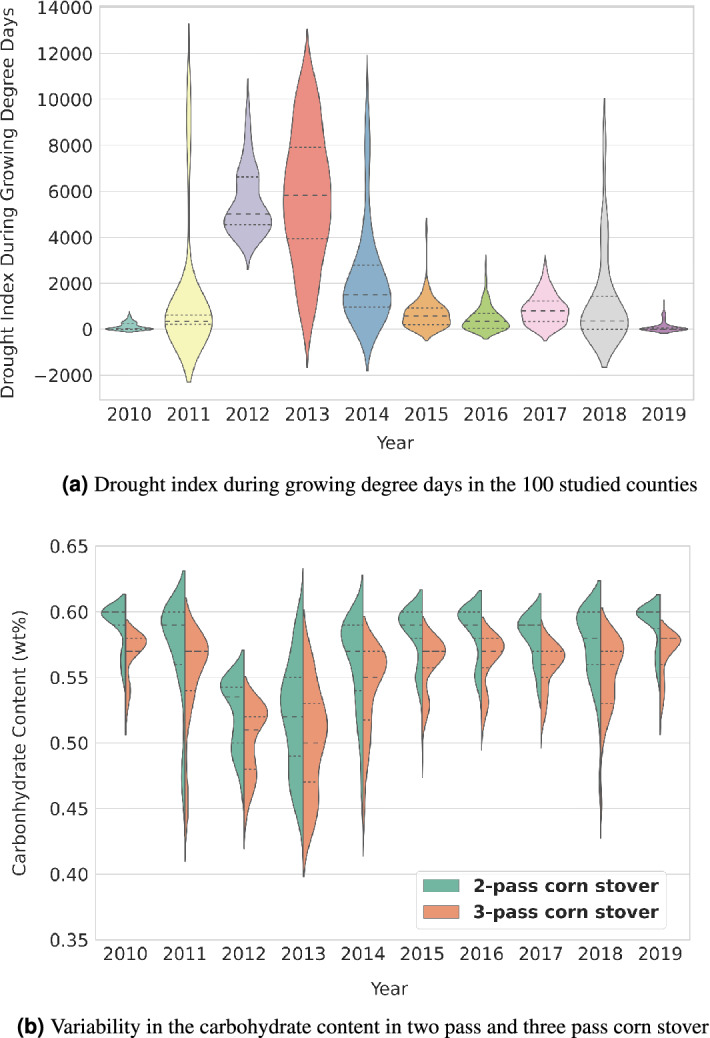
Variability in drought index and carbohydrate contents during 2010–2019.

Extreme weather events, such as drought, have increased in frequency over the last 30 years and are expected to continue to increase^22^. To better understand drought patterns, the University of Nebraska-Lincoln developed the Drought Severity and Coverage Index (DSCI) to quantify drought levels into categories: D0 (abnormally dry) through-D4 (exceptional drought), which can be aggregated into a cumulative drought index (ADSCI) to show drought severity over n weeks or growing degree days^23^. The DSCI are mapped at a county level weekly and provide the resulting data through the U.S. Drought Monitor. Figure 1a shows the DSCI during growing degree days in 100 counties in Kansas, Nebraska and Colorado from 2010–2019. It is noteworthy that the range of variation in the drought index was greater in 2012 and 2013 as compared to other years and, moreover, the average values for these 2 years were higher. 2012, in particular, was a significant nationwide drought year in the United States responsible for $30 billion in losses and a 27% yield reduction for corn grain^24^. Feedstock composition comprising carbohydrate contents are also low and highly variable in these 2 years, as observed in Fig. 1b. In order to handle biomass with higher quality variability, a more robust and advanced supply system is required. Therefore, if the supply chain configuration is optimized based on weather information from years such as 2012 and 2013, the 10-year total supply chain cost is likely to be higher.

### Quality variability

It has been largely recognized that variability in feedstock quality, such as carbohydrate, ash, and moisture content, affect the maximum theoretical product yield for a biofuel conversion process^25^. In addition, inconsistent biomass qualities, such as low carbohydrate levels and high ash content can significantly increase operational costs and decrease overall plant operating effectiveness, mainly by causing downtime and equipment wear during pre-processing operations^26^. Lower biomass carbohydrate and higher ash content also has a negative impact on theoretical ethanol yield in the biofuel conversion process and increased non-convertible materials^27,28^. Similar to yield variability, biomass qualities can also be greatly affected by drought. Figure 1b presents the variability in carbohydrate content for two harvest methods-in two-pass and three-pass, for corn stover in 100 counties in Kansas, Nebraska, and Colorado. 2012 and 2013 have some of the lowest average carbohydrate contents aligning with the high drought indices for these same years. The fundamental mechanics for plants during drought stress are complex. Drought tolerance and response differs by plant type, species, and genotype^29,30^. Increased cell wall elasticity and extractability of cell wall ultrastructure components have been observed in pine under water stress^31,32^. Plant drought responses also include the accumulation of compatible solutes “small, soluble organic molecules like monomeric sugars and proline” to adjust cell water potential^33–35^. It has even been proposed that these small metabolites are prioritized, and the cellulose sacrificed, in order to support osmotic adjustment during drought^36^; however, some studies have shown hemicellulose to be stable or increase under drought conditions exemplifying the complexity of plant-drought interactions and the importance of understanding the severity and duration of drought condition^2,37,38^. Increased extractive components, including soluble sugars, and significantly lower levels of structural sugar such as glucan and xylan have been found in drought-stressed Miscanthus, corn stover, and switchgrass^2,39,40^. However, the negative impact of yield loss and potential decrease in convertible carbohydrates for biofuel conversion may be offset by the fact that some drought-stressed crops also have lower recalcitrance levels. One study demonstrated the changes to the lignin content distribution in the cell wall for corn stalks under water stress impacting cell wall degradability^41^. This decrease in recalcitrance, must be balanced by understanding the negative impact of fermentation inhibitors observed some drought stressed plants like switchgrass^39,40^.

**Figure 2:**
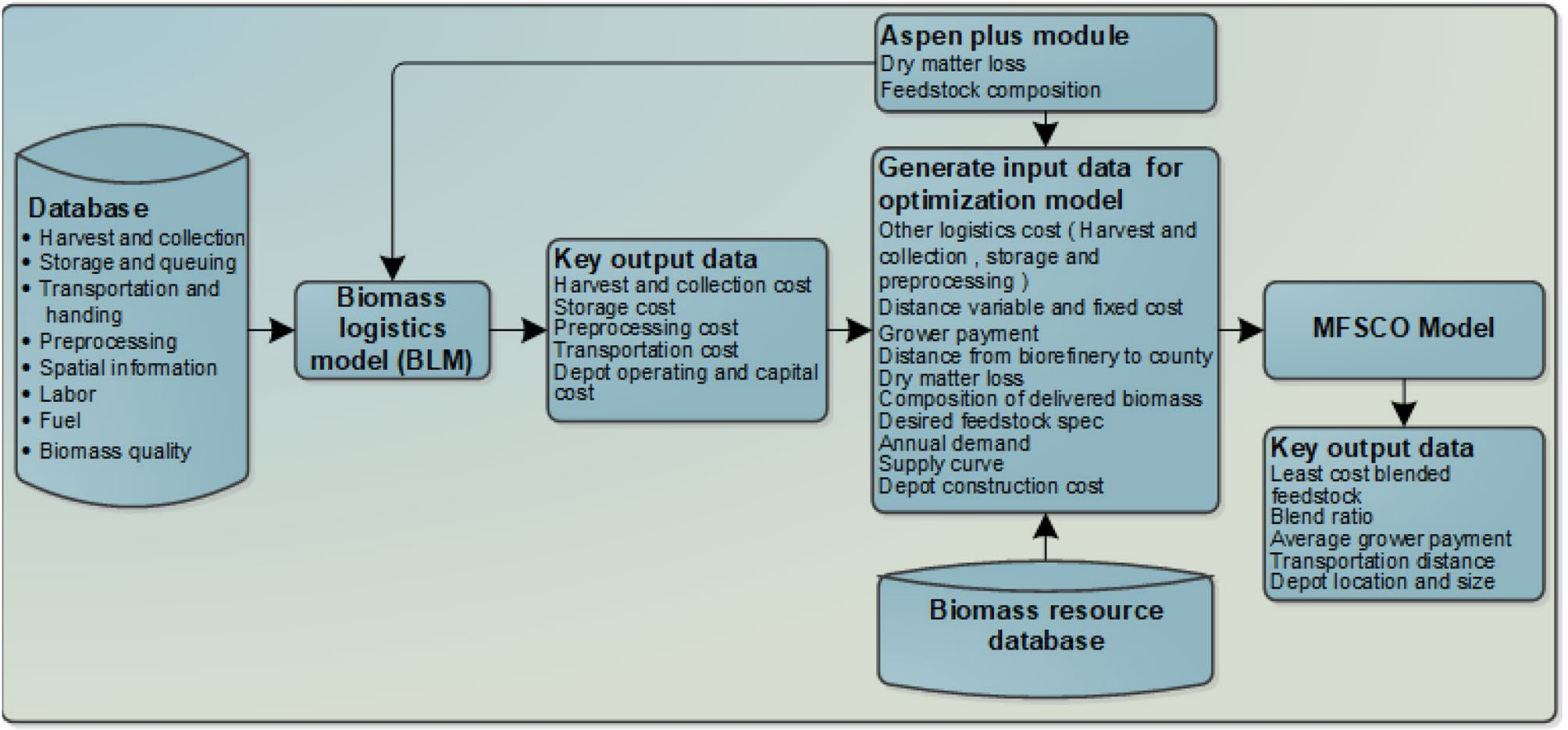
Data flow among models. Biomass logistics model and MFSCO model are utilized in this study.

This study presents a novel biofuel supply chain optimization framework (Fig. 2) that considers yield and biomass quality variability over 10 years and shows the importance of incorporating spatial and temporal variability in supply chain design. The paper is organized as follows: in “Methods” section, we present the methods include biomass variability data collection methodology and the developed model framework are presented; in “Results” section, we present key results such as impacts and importance of incorporating spatial and temporal variability of biomass yield and quality are presented; in “Discussion” section, we provide discussion highlighting the main takeaways based on our findings and potential applications of the developed model.

## Methods

### Biomass variability data collection

#### Yield variability

The primary biomass considered in this study is corn stover. Corn stover yields are closely linked to weather factors, especially precipitation and drought events occurring during growing season^2,42^. Water stress caused by low precipitation and drought can reduce crop yields and can even alter the plant cells and the overall chemical compositions^2,43^. Emerson and Hoover^2^ conducted a study to assess the effects of drought on feedstocks yields and found significantly lower yields in some areas of severe drought. Therefore, in this study, we considered factors of precipitation and drought index during the growing season to estimate corn stover yields.

The yield models used in this study were developed based on corn stover samples and data collected from the Regional Feedstock Partnership project, from year 2009 to 2012^44,45^. Corn stover samples selected for inclusion were from two treatments, each with four replicate field plots, which were the ones that best matched the tillage and management practices leading to the base case 2019 yield that we are using from 2016 Billion-ton report^46^; however, the 50% residue removal likely caused too much variability in the yields to form a decent regression. Therefore, the same treatments were selected except for the residue removal, which was 100%. The corn stover that was included was collected from a conventional tillage practice and a no till practice. Other factors were kept consistent, including the fertility, plant population, and row spacing.Dry biomass for the corn stover residue was determined from the subsample of a dual-stream grain and stover combine. The yield models are presented below:

1 $$\begin{aligned} \log _{10}(Yield_{CT})= & {} -0.60 +(-0.0003\times GS_{ADSCI} )+(-0.0021\times GS_{Precip} )+0.0020\times GDD \end{aligned}$$

2 $$\begin{aligned} Yield_{NT}= & {} 6.76+(-0.0002\times GS_{ADSCI} )+(-0.0008\times GS_{Precip})+0.0020\times GDD \end{aligned}$$

where *CT* stands for corn stover under conventional tillage, *NT* stands for corn stover under no- tillage, $GS_{ADSCI}$ is the Accumulative Drought Severity and Coverage Index (ADSCI) during the growing season. $GS_{Precip}$ is the accumulative precipitation during the growing season, and *GDD* is the total days from the planting date to last frost-free day.The data used in the multiple linear regressions was assessed for normality and homogeneity of residuals. Yield data from the control treatment was log transformed to meet the assumptions of linear regression. The yield data for the no till treatment data set did not require transformation to meet the assumptions of linear regression.

To apply the yield models to estimate corn stover yields in the studied region, the DSCI data for each year from 2010–2019 and each studied county was downloaded from the United States Drought Monitor website developed by the University of Nebraska^47^. Precipitation data was downloaded from website of the National Centers for Environmental Information^48^. Some counties do not have weather records in some years, and for these counties, the average data for the adjacent counties were used to determine the weather.

Since many studies have indicated that tillage system could significantly impact corn stover yield, tillage class was considered in this study^49,50^. As discussed above, two yield models were developed for different tillage classes, conventional tillage (CT) and no-till (NT), and were applied to predict biomass yield for each county in each year. For each county, the acreage of CT and NT was decided based on the query from 2016 Billion-ton report^46^ dataset in year 2019. It is assumed that for each year, the acreage of CT and NT in each county remains the same with year 2019. The yields for each county and each year were predicted using the above-presented regression models. This study requires quantification of yield variability from year to year. The projected corn stover yield reported in the 2016 Billion-ton report^46^ for year 2019 was selected as the base yield for a county. The yield from 2010–2019 of a county is determined by varying base yield. This variation of base yield is quantified by measuring variation of yield calculated from Eqs. (1) and (2).

#### Biomass supply variability

This study requires quantification of corn stover supply variability from year to year at different locations. The projected corn stover yield reported in 2016 Billion-ton study for the year 2019 is selected as base supply for a county. This base supply was varied based on percentage of change in the yield from year 2019 to 2010 for all locations at the county levels in the studied region. A step by step process to determine supply variability of a given location is described below:

1. Use the 2016 Billion-ton projected supply for 2019 as the base supply.
2. Determine biomass supply of 2018 in the same location,
  - Determine percentage increase or decrease in yield of year 2018 from year 2019 in the same location based on model presented in Eqs. (1) and (2).
  - Apply this percentage change to determine biomass supply of 2018.
3. Repeat Step-2 to determine biomass supply for 2010–2017.

#### Composition variability

Drought or water stress is also a key factor that impacts a plant’s chemical compositions. For instance, Al-Hakimi^43^ found significantly lower cellulose and lignin content in soybean plants caused by drought events. Similar findings were also reported in the study conducted by Emerson and Hoover^2^. Drought negatively impacted feedstock qualities and the theoretical ethanol yields. Therefore, in this study, we included precipitation and drought index during the growing season to predict corn stover qualities, including glucan, xylan, and lignin content. These chemical compositions were chosen to be consistent with the annual State of Technology (SOT) report^51^. The models used to predict the chemical compositions were trained using the same dataset collected from conventional tillage treatment and no-till treatment that was part of the Sun Grant/DOE Regional Feedstock Partnership project, from year 2009 to 2012 in Boone County, Iowa as described above. Like the yield prediction models, the chemical composition predicting models were developed for crops under CT and NT classes, and are presented as below:

3 $$\begin{aligned} Glucan_{CT}&= 35.64+(-0.0008\times GS_{ADSCI} )+(0.0003\times GS_{Precip}) \end{aligned}$$

4 $$\begin{aligned} Glucan_{NT}&= 35.25+(-0.0008\times GS_{ADSCI})+(-0.0012\times GS_{Precip}) \end{aligned}$$

5 $$\begin{aligned} Xylan_{CT}&= 20.65+(-0.0006\times GS_{ADSCI})+(0.0002\times GS_{Precip}) \end{aligned}$$

6 $$\begin{aligned} Xylan_{NT}&= 20.65+(-0.0004\times GS_{ADSCI})+(0.0004\times GS_{Precip}) \end{aligned}$$

7 $$\begin{aligned} Lignin_{CT}&= 16.29+(-0.0006\times GS_{ADSCI})+(0.0008\times GS_{Precip}) \end{aligned}$$

8 $$\begin{aligned} Lignin_{NT}&= 16.97+(-0.0005\times GS_{ADSCI})+(0.0003\times GS_{Precip}) \end{aligned}$$

where *CT* stands for corn stover under conventional tillage, *NT* stands for corn stover under no- tillage, $GS_{ADSCI}$ is the ADSCI during the growing season; $GS_{Precip}$ is the accumulative precipitation during the growing season. The data used in the multiple linear regressions was assessed for normality and homogeneity of residuals. All compositional models met the underlying assumptions of linear regression. In addition, linear models have been successfully applied in similar studies of switchgrass^52^. In this recent publication, switchgrass was grown in multiple locations in the U.S. and precipitation and drought had strong relationships with glucan, xylan, and lignin content. We hypothesize that glucan, xylan, and lignin content of corn stover grown in multiple locations would also have strong relationships with precipitation and drought in unirrigated fields. In addition, it is anticipated that the coefficients might change but the trends may stay the same. Given the complexity of real-world systems this would need to be tested with additional field trials; however, this is beyond the scope of the current paper and will be the topic of future research.

Initial compositions of three-pass and two-pass corn stover were predicted using the above equations and weather data for each county and each year. Part of the ash in corn stover is intrinsic inorganics in the plants, which is determined by environmental conditions during the growing season; while the other part of the ash is caused by soil introduction during harvesting, and is closely related to the harvesting method used^53^. Currently, there is no available tool to predict physical ash of corn stover based on the weather conditions. Therefore, it was assumed that physical ash content for corn stover was 5%, the ash introduced by two-pass and three-pass harvesting was about 2% and 6%.

The moisture content of corn stover greatly depends on the weather conditions around the time it was harvested. For instance,Womac et al.^54^ predicted moisture based on environmental conditions, utilizing rain fall, air relative humidity and evaporation for different harvesting time and methods. We do not have experimental data that can quantify the correlation between moisture in the harvested biomass with precipitation and drought index. Hence, moisture variability is not scoped within this study.

### Modeling framework

An optimization model is developed to demonstrate supply system variability and identify least cost supply chain. This model is an extension of previous model utilized to determine least cost supply chain model^55^. This model determines optimal depot locations and size considering the variability of yield, biomass supply and quality parameters encountered in these sources from year 2010–2019. The optimization model has two types of decision variables: strategic and operational decision variables. Strategic decision variables aim to identify where to locate the depots, and at what sizes. This is strategic decision variables because once the depots are constructed, they cannot be changed from year to year. Operational decision variables aim to identify where to source biomass, which type, and what quantity to meet biorefinery’s feedstock demand. This decision can change from year to year. The objective of the optimization model is to minimize the stretegic decision cost (e.g. depot construction cost) and operational decision cost (e.g delivered feedstock cost) over 2010–2019 for given variability in yield, biomass supply and quality parameters in the studied region. The optimization model is formulated as a mixed integer programming model (MILP). The following notations are used in the MILP model.

**Sets**

*F*: Feedstock sets
*P*: Feedstock price sets
*H*: Field site sets
*I*: Feedstock storage location sets
*J*: Candidate locations of depot sets
*K*: Depots size sets
*B*: Biorefinery location sets

**Biomass specific parameters**

$$l_{ift}:$$
Dry matter loss percentage (%) of feedstock type *f* at location *i* in the time period *t*
$$c_{ijft}:$$
Carbohydrate percentage (%, dry basis) in feedstock type *f* at location *i* in the time period *t*
$$a_{ift}:$$
Ash percentage (%, dry basis) at location *i* in feedstock type *f* in the time period *t*
$$S_{ifpt}:$$
Available supply (%, dry basis) of feedstock *f*, at location *i* at price *p* in the time period *t*
$$m_{ift}:$$
Moisture (%, wet basis) in the harvested biomass from location *i* for feedstock type *f* in the time period *t*
*c:* Required carbohydrate percentage (%) in the blended feedstock
$$\bar{a}:$$
Required ash percentage (%) in the blended feedstock
$$\phi:$$
Average ratio (%) of two-pass and three-pass harvested corn stover yield

**Economic parameters**

*Field side*

$$g_{ifpt}:$$
Price ($/dry Mg) paid to farmer before harvesting at location *i* for feedstock *f* at purchase price *p* point in the time period *t*
$$v_{ift}:$$
Cost ($/dry Mg) to harvest and collect biomass at field side *i* for feedstock *f* in the time period *t*
$$s_{ift}:$$
Cost ($/dry Mg) to store biomass at field side *i* for feedstock *f* in the time period *t*

*Field side to depot*

$$e_{ijf}:$$
Cost ($/dry Mg) to transport from location *i* to depot *j* for feedstock type *f* in the time period *t*
$$u_{jk}:$$
Depot at location *j* with capacity *k*
$$v_{jk};$$
Depot construction cost ($/dry Mg) at location *j* with capacity *k*
$$r_{jft}:$$
Cost ($/dry Mg) to pre-process biomass *f*, at location *j* in the time period *t*
$$s_{jft}:$$
Cost ($/dry Mg) to store at depot *j* for feedstock *f* in the time period *t*
$$h_{jft}:$$
Material handling cost ($/dry Mg) at depot *j* for feedstock *f* in the time period *t*
$$\mu _{j}:$$
Depot capacity utilization factor (%) at location j

*Depot to biorefinery reactor throat*

$$D_{j}:$$
Annual demand ($/dry Mg) of dry biomass at location *j*
$$e_{ijft}:$$
Cost ($/dry Mg) to transport pellet from location *i* to location *j* for feedstock type *f* in the time period *t*
$$s_{jft}:$$
Cost ($/dry Mg) to store pellet at biorefinery *j* for feedstock *f* in the time period *t*
$$h_{jft}:$$
Cost ($/dry Mg) to material handling at biorefinery *j* for feedstock *f* in the time period *t*
$$b_{jft}:$$
Cost ($/dry Mg) to blend at biorefinery *j* for feedstock *f* in the time period *t*
$$\upsilon:$$
Ash disposal cost ($/dry Mg)
$$\iota:$$
Carbohydrate dockage ($/dry Mg)

The strategic decisions variables defined by $W_{jk}$ is a binary variable to decide depot location *j* with capacity *k*. The operations decisions variables defined by $X_{ijfp}$, $Y_{ijft}$ , $Z_{ifp}$ and $\pi _{jt}$. The variable $X_{ijfp}$ is a continuous variable that decides the procured biomass type *f* from location *i* to *j* at purchase price *p* in the time period *t*. The variable $Y_{ijft}$ tracks the flow of carbohydrate content from location *i* to *j* for feedstock type *f* in the time period *t*. $Z_{ifp}$ is a binary variable to decide on a specific farm gate price *p* of biomass *f* from location *i* in the time period *t*. Finally $\pi _{jt}$ is a continuous variable used to used to track shortage of carbohydrate content at location *j* in the time period *t*.

The objective function of the optimization model minimizes feedstock supply chain cost 10-year period and is defined by Eq. (10)

9 $$\begin{aligned} \min \sum _{j\in J}\sum _{k\in K}\eta _{jk}Q_{jk}W_{jk}+\frac{1}{T}\left[ \sum _{i\in {H}}\sum _{j\in {I}}\sum _{f\in {F}}\sum _{p\in {P}}\sum _{t \in {T}} \left( g_{ifpt}+v_{ift}+s_{ift}\right) X_{ijfpt} +\sum _{i\in {I}}\sum _{j\in {J}}\sum _{f\in {F}}\sum _{p\in {P}}\sum _{t \in {T}} \left( e_{ijft}+h_{jft}+r_{jft}+s_{jft}\right) X_{ijfpt}\right] \end{aligned}$$

10 $$\begin{aligned} +\frac{1}{T}\left[ \sum _{i\in {J}}\sum _{j\in {B}}\sum _{f\in {F}}\sum _{p\in {P}}\sum _{t \in {T}}\left( e_{ijft}+s_{jft}+h_{jft}+b_{jft}\right) X_{ijfpt}+\sum _{j\in {B}}\sum _{t \in {T}} \iota \pi _{jt}+\sum _{i\in {J}}\sum _{j\in {B}}\sum _{f\in {F}}\sum _{p\in {P}}\sum _{t \in {T}}\left( a_{ijft}-\bar{a}\right) X_{ijfpt}\right] \end{aligned}$$

The first term in the objective function represnets the cost of locating a depot at location *j* with capacity *k*.The second term in the objective function is the average farm gate cost i.e., the sum of feedstock procuring, harvesting, and storing at the farmgate. The third term in the objective function is the average cost of transporting from the field side to the depot, handling and queuing, preprocessing, and storing of biomass. The fourth term in the objective function is the average cost of transporting biomass from the depot to the biorefinery, storage, handling and queuing, and blending cost at biorefinery. Finally, the fifth and sixth terms are the average carbohydrate and ash dockage cost.

The optimization model has various constraints defined by Eqs. (11–26).

#### Resource availability constraints

The constraint (11) states that biomass availability is limited from a supply location $i\in H$ to depot location $j \in I$ with grower payment $p\in P$ the time period *t*. Constraint (12) select a single contract price from the supply curve of the location $i\in H$ at the time period *t*. Let define set $f=\{f_{1},f_{2}\}$, where $f_{1}$ represents three-pass corn stover, $f_{2}$ represents two-pass corn stover. Constraint (13) limits the total availability of two-pass and three-pass corn stover from a supply location a specific farm gate price at time period *t*.The details of these constraints are described in a previous study^55^.

11 $$\begin{aligned}&X_{ijfpt}\le S_{ifpt}Z_{ijfpt} \quad \quad \forall i \in H, \forall j \in I, \forall f \in F,\forall p \in P, \forall t \in T \end{aligned}$$

12 $$\begin{aligned}&\sum _{p\in P}Z_{ijfpt}=1 \quad \quad \forall i \in H, \forall j \in I, \forall f \in F, \forall t \in T \end{aligned}$$

13 $$\begin{aligned}&X_{ijf_{1}pt}+\frac{X_{ijf_{2}pt}}{\phi } \le S_{if_{1}pt} \quad \quad \forall i \in H, \forall j \in I, \forall p \in P, \forall t \in T \end{aligned}$$

#### Biomass and carbohydrate flow balance constraints

Constraint (14) represents the mass balance flow constraints at field storage. Constraint (15) represents the mass flow balance constraints at depots. Constraint (16) represents the flow conservation constraints for a specific biomass at a depot for a specific type of biomass. Constraint (17) represents the carbohydrate mass balance constraints at depots. Constraint (18) represents the carbohydrate mass balance constraint at depots for a specific type of biomass.

14 $$\begin{aligned}&\sum _{i= j}(1-l_{ift})X_{ijfpt}-\sum _{i\in J}X_{jifpt}= 0 \quad \quad \forall j \in I, \forall f \in F,\forall p \in P, \forall t \in T \end{aligned}$$

15 $$\begin{aligned}&\sum _{i \in j}\sum _{f \in F} \sum _{p \in P}(1-l_{jft})X_{ijfpt}-\sum _{i \in B}\sum _{f \in F} \sum _{p \in P}X_{jifpt}= 0 \quad \quad \forall j \in J, \forall t \in T \end{aligned}$$

16 $$\begin{aligned}&\sum _{i\in I}(1-l_{jft})X_{ijfpt}-\sum _{i\in B}X_{jifpt}= 0 \quad \quad \forall j \in J, \forall f \in F,\forall p \in P, \forall t \in T \end{aligned}$$

17 $$\begin{aligned}&\sum _{i \in j}\sum _{f \in F} \sum _{p \in P}(1-l_{jft})c_{ijfpt}X_{ijfpt}-\sum _{i \in B}\sum _{f \in F} \sum _{p \in P}Y_{jift}= 0 \quad \quad \forall j \in J, \forall t \in T \end{aligned}$$

18 $$\begin{aligned}&\sum _{i\in I}(1-l_{jft})c_{ijfpt}X_{ijfpt}-\sum _{i\in B}Y_{jift}= 0 \quad \quad \forall j \in J, \forall f \in F,\forall p \in P, \forall t \in T \end{aligned}$$

#### Depot location and capacity constraints

Constraints (19) and (20) are the maximum and minimum capacity utilization of a depot. Constraint (21) states that maximum one depot can be located.

19 $$\begin{aligned}&\sum _{i \in I}\sum _{f \in F} \sum _{p \in P}(1-l_{jft})X_{ijfpt}-\sum _{k \in K}Q_{jk}W_{jk}\le 0 \quad \quad \forall j \in J, \forall t \in T \end{aligned}$$

20 $$\begin{aligned}&\sum _{i \in I}\sum _{f \in F} \sum _{p \in P}(1-l_{jft})X_{ijfpt}-\sum _{k \in K}\mu Q_{jk}W_{jk}\ge 0 \quad \quad \forall j \in J, \forall t \in T \end{aligned}$$

21 $$\begin{aligned}&\sum _{k \in K}W_{jk}\le 1 \quad \quad \forall j \in J \end{aligned}$$

#### Total feedstock and carbohydrate demand constraints

Constraint (22) enforces that total feedstock demand of a biorefinery is satisfied. Constraint (23) states that total carbohydrate content requirement (*c*) in the total feedstock are met.

22 $$\begin{aligned}&\sum _{i \in J}\sum _{f \in F} \sum _{p \in P} X_{ijfpt}= D_{jt} \quad \quad \forall j \in B, \forall t \in T \end{aligned}$$

23 $$\begin{aligned}&\sum _{i \in J}\sum _{f \in F} \sum _{p \in P}Y_{ijft}+\pi _{jt}\ge cD_{jt} \quad \quad \forall j \in B, \forall t \in T \end{aligned}$$

#### Other constraints

Finally, constraint (24) states that this decision variables are continuous and non-negative, and constraints (25) and (26) state that they are binary decision variables.

24 $$\begin{aligned}&X_{ijfpt}, Y_{ijft}, \pi _{jt}\ge 0 \end{aligned}$$

25 $$\begin{aligned}&Z_{ifpt}\in \{0,1\} \quad \quad \forall i \in H, \forall f \in F,\forall p \in P, \forall t \in T\ \end{aligned}$$

26 $$\begin{aligned}&W_{jk}\in \{0,1\} \quad \quad \forall j \in J, \forall k \in K \end{aligned}$$

#### Solution approach

The optimization model defined by Eqs. (10–26) is solved by commercial solver CPLEX with C++ API. CPLEX defualt setting can solve the model wihin 60 seconds using desktop computer (Intel(R) Xeon(R) Gold 6252 CPU @ 2.10 GHz processor) and Windows operating system.

### Data flow between biomass logistics model and MFSCO model

Input cost for unit operations of biomass logistics (e.g. harvest and collection cost, pre-processing etc.) used in this study is determined by the Biomass Logistics Model (BLM)^56^. Figure 2 shows the data flow among models utilized in this study.

## Results

### Importance of incorporating spatial and temporal variability of biomass yield and quality in feedstock supply chain design consideration

A comparative case study was conducted utilizing a Multi-Feedstock Supply Chain Optimization (MFSCO) model to understand the importance of incorporating spatial and temporal variability into the feedstock supply chain design consideration. The MFSCO models a distributed depot-based supply chain system^55^ to handle potential feedstock supply uncertainty by providing access to a larger supply shed. This distributed depot-based supply chain system deploys biomass depot where different raw biomass are converted to pellets via two stage size reduction process and high moisture pelleting.

This study provided an high resolution analysis based on a hypothetical biorefinery annual feedstock demand of 653,225-Mg/year. The physical location of this hypothetical biorefinery is at Sheridan County in Kansas, USA. Feedstock demand can be met by corn stover harvested via a two-pass and three-pass harvesting method. High resolution analysis utilizes resources, quality constraint within the MFSCO model. Primary output from the MFSCO model is the feedstock cost, optimal ratio of feedstock, and supply chain design decisions.

In this study, we ran an optimization model under different scenarios to understand supply system variability measured by optimal depot location, size, feedstock blend and cost. From the modeling results, we compared depot locations, sizes and delivered feedstock cost under single year spatial variability of biomass yield and composition. Table 1 shows that depot locations, sizes and numbers are different in year from 2010–2019. For example, depot locations in the year of 2019 is not same as 2018. Moreover, year 2011–2012 and 2019 required three depots where the rest of the years utilized two depots. This implied that optimal supply chain decision based on single year variability would not be optimal for another year. In reality once depots are constructed, they can’t be changed. Hence, depots need to be identified economically to handle the variability in biomass sources and quality parameters encountered in these sources over a 10-year period.

**Table 1:** Comparison of delivered feedstock cost, numbers of depot location and size if supply chain design decision is made based on single year vs 10 year temporal and spatial variability in biomass yield and quality.

Scenario	Delivered feedstock cost ($/dry Mg)	10-year average delivered feedstock cost ($/dry Mg)	Size	County	State
Scenario 1 (2010-based)	$78.11	$95.28	272,100	Franklin	NE
			430,825	Phelps	NE
Scenario 2 (2011-based)	$82.26	$94.03	249,425	Clay	NE
			362,800	Franklin	NE
			90,700	Gosper	NE
Scenario 3 (2012-based)	$109.36	$97.41	226,750	Jefferson	NE
			272,100	Platte	NE
			204,075	Richardson	NE
Scenario 4 (2013-based)	$97.19	$98.81	294,775	Nemaha	KS
			204,075	Jefferson	NE
			204,075	Johnson	NE
Scenario 5 (2014-based)	$85.60	$92.72	272,100	Furnas	NE
			430,825	Hamilton	NE
Scenario 6 (2015-based)	$86.04	$96.26	362,800	Clay	NE
			340,125	Phelps	NE
Scenario 7 (2016-based)	$83.14	$101.16	226,750	Haskell	KS
			476,175	Gosper	NE
Scenario 8 (2017-based)	$83.30	$98.44	181,400	Haskell	KS
			521,525	Phelps	NE
Scenario 9 (2018-based)	$80.87	$95.09	181,400	Hall	NE
			521,525	Phelps	NE
Scenario 10 (2019-based)	$82.94	$94.23	204,075	Clay	NE
			226,750	Franklin	NE
			272,100	Gosper	NE
Scenario 11 (10-year-based)	$92.37	$92.37	430,825	Clay	NE
			272,100	Phelps	NE

To analyze the economic impact of single year variability decision vs multi-year variability, we compared the average delivered cost of supply chain design for the year 2010–2019 if depot locations, sizes and quantity were decided based on multi-year variability of biomass yield and composition. Table 1 shows optimal depot location, size and quantity if supply chain design were decided based on weather and precipitation variability observed during the year from 2010–2019. The 10-year average delivered cost is $92.61/dry Mg. If we compared the 10-year average delivered cost with the delivered cost of each year of Table 1, the results showed that delivered cost is underestimated in most of the years from 2010–2019. This implied that a biorefinery should incorporate spatial and temporal variability in biomass quality and yield in feedstock supply chain design consideration for accurate assessment of feedstock supply chain cost.

**Figure 3:**
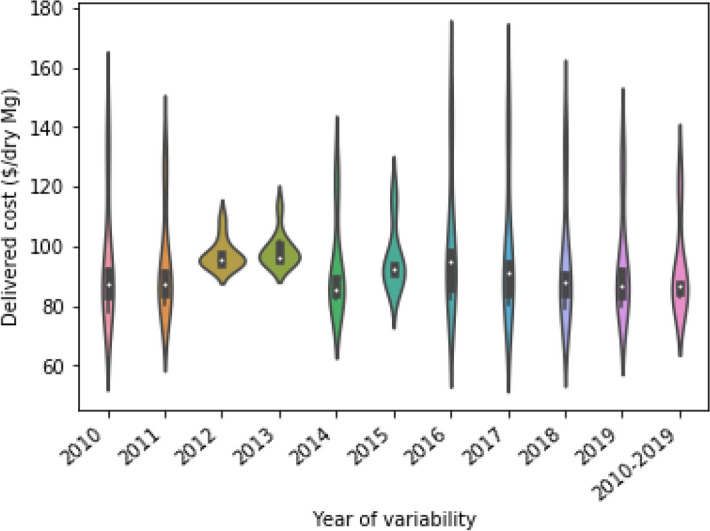
Variability in delivered cost.

We further investigated the negative impact of ignoring spatial and temporal variability on feedstock supply chain design by comparing the total delivery costs of scenarios that were optimized based on different single years. Figure 3 shows the distribution of delivered cost over 10 years if depot location and size is determined based on single year variability and 10 years variability. As shown in the figure, if the supply chain is optimized considering only the biomass yield and quality in a single year, the biomass delivery cost would vary largely. For example, the distribution plot under 2016 in the figure implied that if depots are identified based on weather and precipitation index of 2016, the delivered cost is expected to be highly variable across the years. Similar distribution was observed in the delivered cost for most year if depots were identified based on weather and precipitation index of single year. However, if depot locations and sizes were optimized over the 10-year period (2010–2019), the median delivered cost of the 2010–2019 scenario was lower than for other scenarios. The shape of the distribution throughout the 2010–2019 scenario also indicated that delivered cost was highly concentrated around the median.

**Figure 4:**
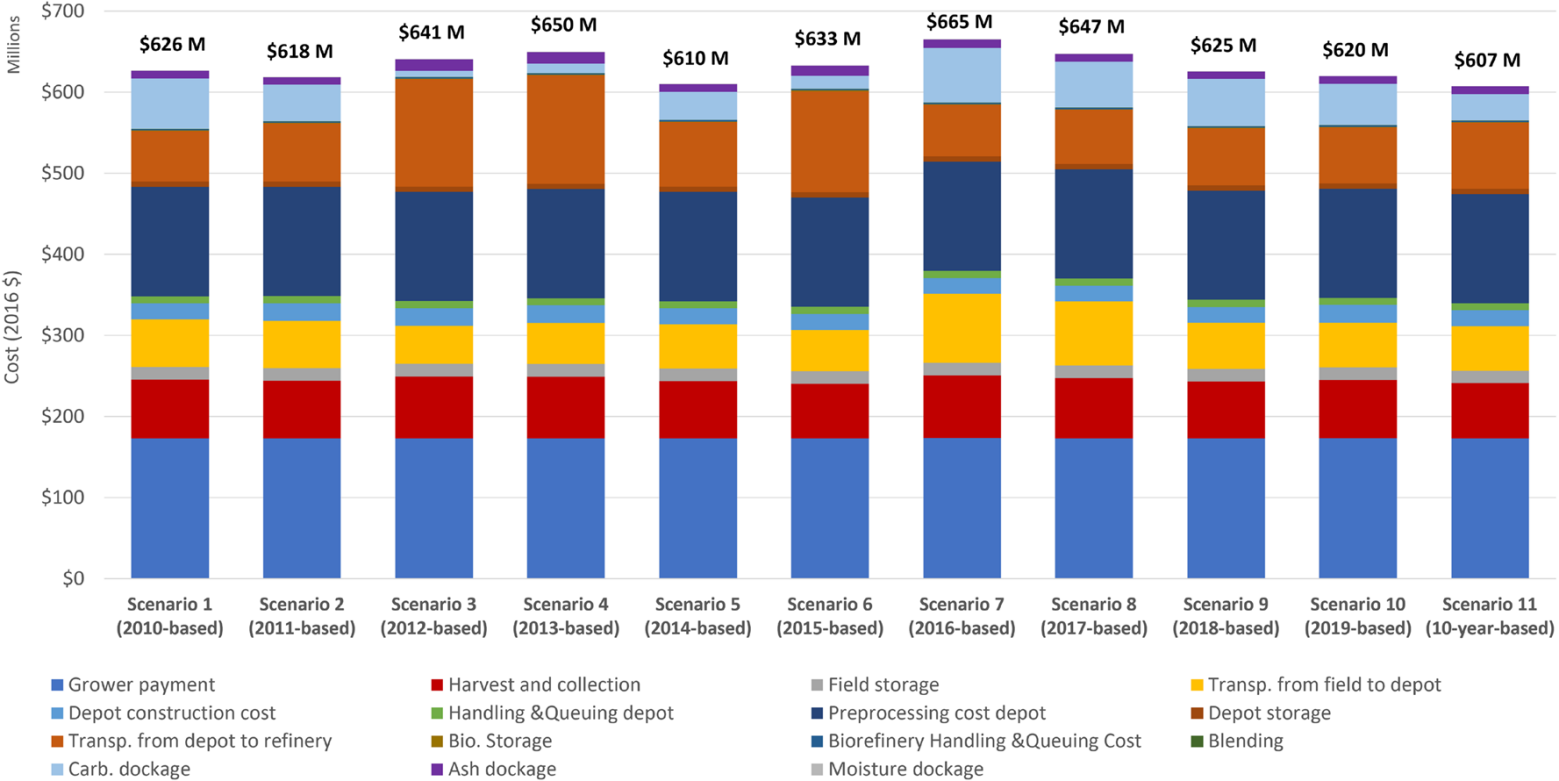
Total cost if depot location, size and quantity are decided based on single year variability or multi-year variability.

Figure 4 shows the 10-year total cost if the depot locations and sizes were decided based single year variability and over 10 years (2010–2019). For example, the Scenario 1 (2010-based) case refers to the scenario that depots are identified based on weather and precipitation index of 2010. The Scenario 11 (2010–2019 based) case in Fig. 4 indicates that depot are identified based on weather and the precipitation index of year 2010 to year 2019. Table 1 shows that depot locations, sizes and numbers are different in the years 2010–2019. Result shows that the base case: Scenario 11 (2010–2019 based) incurs the lowest 10-year average and total delivered cost because depots are located to handle all the variability observed from 2010–2019. Figure 4 shows that in the worst case decision (Scenario 7 (2016-based)), total biomass delivered cost is about $58 M higher compared to the base case. Even in the best case scenario (Scenario 5 (2014-based)), the 10-yr total cost is still $2 M higher than making the decision based on the 10-yr variability.

### Correlation between weather index and supply chain decision

**Figure 5:**
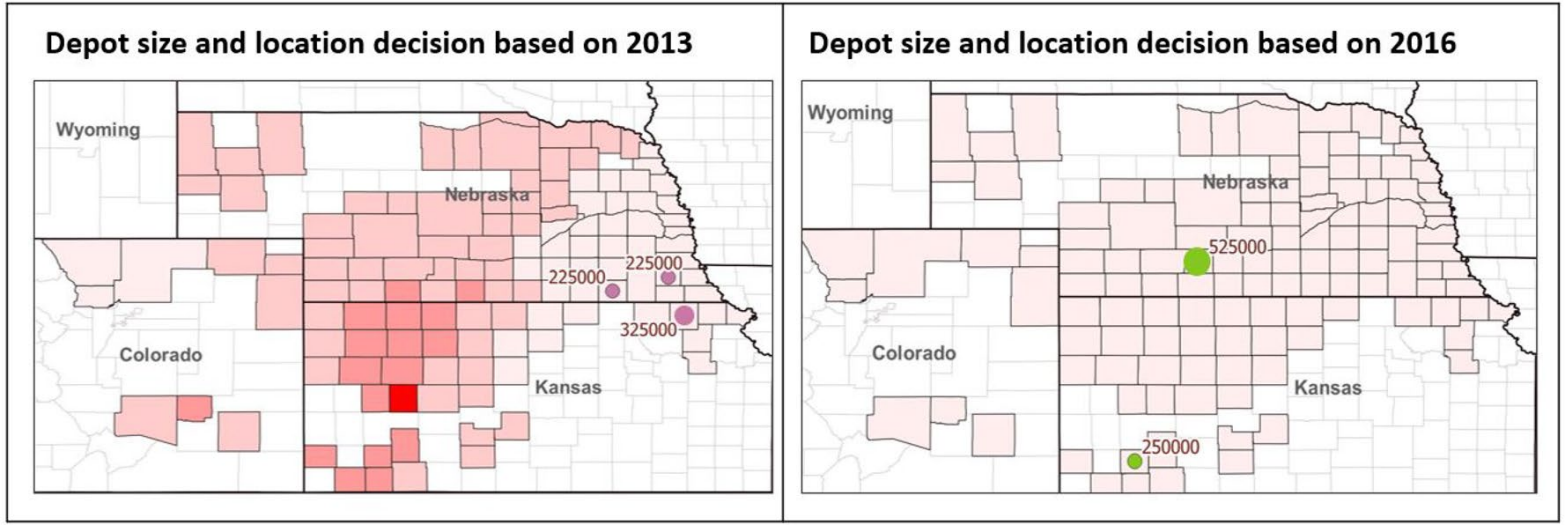
Depot location based on 2013 and 2016’s weather information with drought index map.

Drought variability index shows that 2012 and 2013 are relatively dry years in the studied region stemming from a combination of factors impacting drought conditions, including low precipitation rate and soil moisture, as well as high temperatures and evaporative demand, as shown in Fig. 1a. Drought index in these years are highly variable. Feedstock composition comprising carbohydrate contents are also low and highly variable in these 2 years, as shown in Fig. 1b. To demonstrate the impact of weather-related index on feedstock supply chain decisions, feedstock delivered cost, depot locations and sizes are compared under these three scenarios: (1) optimal depot locations are made to handle the worst-case drought scenario, (2) optimal depot location decisions are made based on one of the best drought scenario; and (3) optimal depot location decisions are made based on 2010–2019 drought scenario. If optimal depot locations are made to handle the worst-case scenario (i.e.variability observed in 2013), three depots would be needed as shown in Table (1) and Fig. 5. If optimal depot location decisions are made based on one of the best case scenario (i.e., variability observed in 2016), only two depots would be needed as shown in Table(1) and Fig. 5. In 2016, drought severity was generally low in all considered counties. To minimize the total biofuel production cost, a 476,175 dry Mg depot is located at Gosper, Nebraska and another 225,750 dry mg depot is located at Haskell, Kansas. If we look at the annual cost breakdown for the 2016-based scenario, it can be observed that the total transportation cost from the field to the depot of the 2016-based scenario is much higher than the other optimization scenarios, which fully illustrates that the location and size based on 2016 is not the best choice. In addition, the significantly higher carbohydrate dockage cost in the 2016-based scenario also suggested that compared to the 10-year based scenario, the biorefinery will face about 109% higher dockage cost when the location is not optimized considering drought years such as 2012 or 2013. If optimal depot location decisions are made based on the variability of 2010–2019, two depots will be needed (Table 1). Variability in optimal depot locations and sizes to handle different drought scenarios calls for robust supply chain decisions to drive down the long-term feedstock supply chain cost.

**Figure 6:**
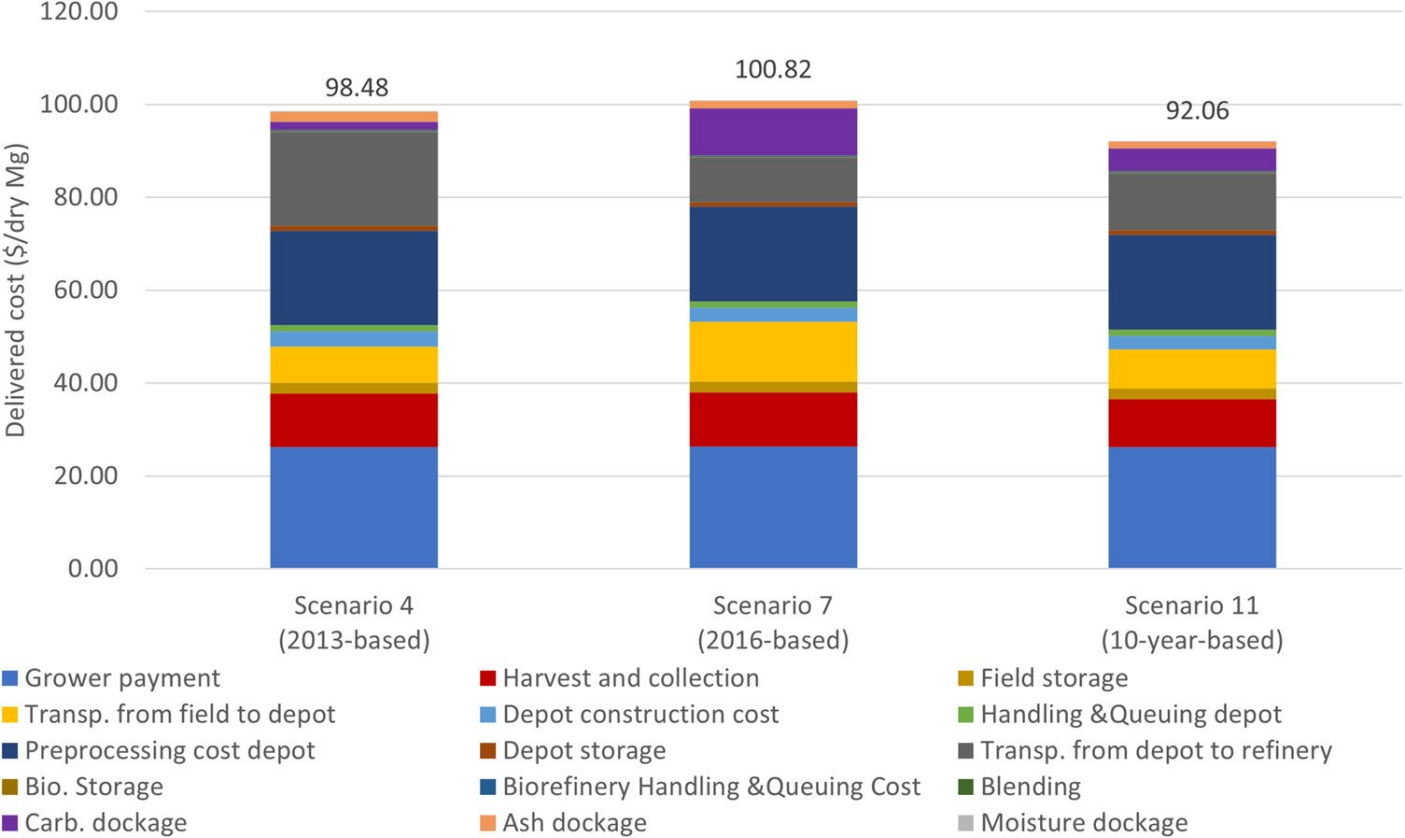
Cost ($/dry Mg) breakdown of scenario 4, 7 and 11 while supply chain is optimized based on single- and multi-year variability.

The 10-year average delivered cost is compared to understand the economic impact of making supply chain decision based on worst drought index, best drought index, and multi-year drought index. For example, three depots are needed to handle the worst case scenario: Scenario 7, the 10-year average delivered feedstock cost is $100.82/dry Mg (Fig. 6). In a scenario like Scenario 4 where the depot locations and sizes are optimized not only to handle the worst-case scenario but also minimize the total cost over the 10 years period, the 10 average delivered feedstock would be $98.48/dry Mg. As shown in the results, the supply chain decisions considering worst case weather index is not necessarily robust, and would result into higher delivered feedstock cost. Since drought severity was generally low in all considered counties in 2016, if the depot locations are determined based on 2016 drought index, it would increase the cost of delivering biomass during the drought year. As a result if the depot locations are determined based on 2016 drought index, average delivered cost is highest among these three scenarios. This result implies that multi-year weather index should be considered within supply chain decision to avoid the risk of a higher delivered cost.

### Sources of variability in the feedstock delivered cost in scenario 11 (2010–2019 based)

Feedstock-delivered cost comprises grower payment, harvesting operations cost, storage cost at field and depot, cost to transport biomass, preprocessing, handling and queuing cost at depot and biorefinery, and dockage—a penalty cost for not meeting feedstock specification. Spatial and temporal variability in biomass yield and quality caused by weather does not affect different cost components evenly. To illustrate the variability in the delivered feedstock cost and how these cost components changes over years, the proportion of different cost components are analyzed from 2010 to 2019. This analysis identified different cost component from year 2010–2019 assuming that the two depots will be deployed in the case study. These two depots are identified considering spatial and temporal variability of yield and quality from 2010–2020, as shown in Table 1.

**Figure 7:**
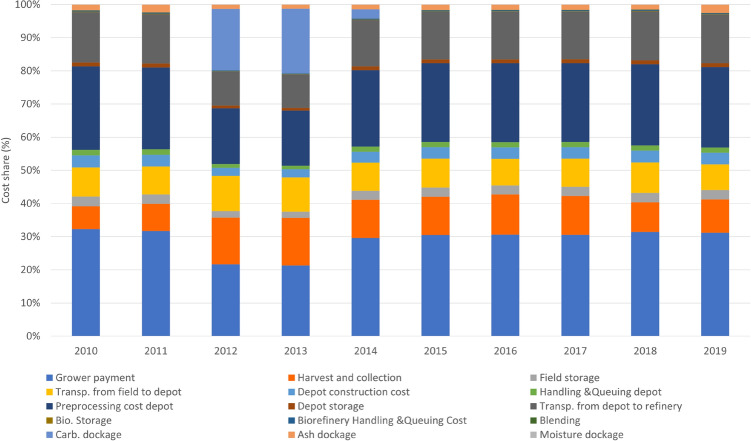
Cost share of each supply chain component in the total feedstock cost in Scenario 11 (2010–2019 based) (supply chain is optimized based on 10-year weather index).

Figure 7 shows the cost share of each supply chain component in the total feedstock cost. Major cost share components are: grower payment, preprocessing cost at depot, harvest and collection cost, transportation cost, and carbohydrate dockage. Figure 7 shows that carbohydrate dockage is a major portion of delivered cost in the year of 2012 and 2013 due to the fact that 2012 and 2013 are relatively dry years in the studied region with low precipitation rates. Since a drought-stressed year incurs lower levels of structural sugar such as glucan and xylan, a carbohydrate dockage (a penalty cost for not meeting carbohydrate specification of feedstock) is observed in these two years. If the proportion of the carbohydrate dockage is converted to $/dry Mg, carbohydrate dockage varies from $0–23.97/dry Mg. In these two years, harvest and collection cost are also higher and contribute more to the total cost share due to the lower biomass yield. If the proportion of the cost is converted to $/dry Mg, harvesting cost varies from $5.6–17.65/dry Mg. Transportation cost is also observed to be higher in the year of 2012 and 2013. If the proportion of the field to depot transportation cost is converted to $/dry Mg, transportation cost from field depot varies from $6.54–12.90/dry Mg.

### Addressing current barriers to attract private investment

One of the current barriers of the cellulosic biorefining industry is it’s inability in attracting private investment. This industry’s inability to balance risk and return is partially blamed for not springing up cellulosic biorefinery across the United States. The uncertain characteristics of the biomass supply and quality imposes supply and quality risk, which ultimately increases the operational risk of a biorefinery. The operational risk brings the uncertainty of producing cellulosic biofuel within a profitable range. Since supply chain planning considering the multi year weather index helps to avoid the risk of higher feedstock cost, it is expected that the variation of cellulosic biofuel cost caused by feedstock cost will be minimized. In order to quantify the risk of higher feedstock cost we computed the probability of exceeding the average delivered cost of $92.37 from the frequency of delivered cost listed in the Fig. 3. It is observed that the probability of exceeding average delivered cost of $92.37 is 20.0% in the case of the supply chain planning considering the multi-year weather index, whereas the probability of exceeding of average delivered cost of $92.37 can be up to 100.0% in the case of supply chain planning with single-year weather index.

## Discussion

Successful biofuel commercialization from agricultural residue and waste does not depend on a single factor. The biofuel industry needs to overcome many barriers including feedstock logistics and supply chain issues. The research aims to address supply chain barriers caused by temporal and spatial variability of biomass yield and quality in order to achieve profitability and competitiveness with the traditional fossil fuels industry. Since weather factors such as drought or water stress is a key factor contributing to the biomass yield and quality variability, this study incorporates the spatial and temporal variability of biomass yield and quality caused by variation of accumulated precipitation and drought severity and coverage index during the growing season. Accumulative precipitation and ADSCI data for Kansas, Nebraska, and Colorado for the year of 2010–2019 were utilized to provide managerial insights on the importance of incorporating spatial and temporal variability of biomass quality and yield within feedstock supply chain planning.

The results show that the 10-year average delivered feedstock cost can be effectively reduced by optimizing the depot location and size based on multi-year variability in biomass yield and quality. For example, case study accomplished in this research shows that if the optimal depot locations and sizes are determined based on variability observed in 2016, average feedstock delivered cost over a 10-year period would be $101.15/dry Mg. However, if depot location and size are determined based on variability observed from year 2010–2019, average feedstock delivered cost over the 10-year period (2010–2019) would be $92.37/dry Mg. In order to minimize long term feedstock delivered cost, it is important that feedstock supply chain decisions are optimized over a longer period considering the variability in biomass sources and quality parameters encountered in biomass sources.

Given a biorefinery optimizes supply chain considering long-term spatial and temporal variability of biomass yield and quality, delivered cost still can vary from year-to-year in different ways. Yield variability from year-to-year changes harvesting efficiency. As a result, it also affects harvesting and collection cost. Since yield variability also affects biomass supply, a biorefinery cannot source biomass from the same locations each year. As a result, field to depot transportation cost changes each year. Similar to yield variability, biomass qualities change due to weather variability. At least three of the 10 years of carbohydrate dockage is applied due to lower biomass carbohydrate content in the feedstock. Thus, the delivered cost changes from year to year due to the change in harvesting and collection cost, transportation cost, and carbohydrate dockage. Other factors (e.g., energy price, labor rate) that could change delivered cost year to year is not scoped within this study.

The conventional practices of supply chain planning of a biorefinery that ignores long term spatial and temporal variability of biomass yield and quality can be a barrier to successful commercialization of biofuels made from renewable biomass and waste resources in different ways. Variability in feedstock quality such as carbohydrate content, ash, and dry-matter loss jeopardize the certainty of feedstock cost and conversion efficiency. Since the minimum fuels selling price (MFSP), feedstock cost, and conversion efficiency are interdependent, this spatial and temporal variability increases the risk of MFSP exceeding the profitable selling price target. This risk may prevent investment in new biorefinery utilizing renewable biomass and waste resources. Since spatial and temporal variability of biomass yield and quality, jeopardize the certainty of MFSP, risk premium or equity cost of financing on an investment in a biorefinery may increase.

Incorporating spatial and temporal variability during biomass supply chain planning via optimization modeling has the potential to overcome the uncertainty of MFSP of biofuels caused by variability in feedstock quality and yield. Since the supply chain decisions are made based on long-term spatial and temporal variability of biomass yield and quality via a multi-period optimization method, the supply chain decisions are robust to handle. As a result, a biorefinery is minimizing risk of underestimating or overestimating of feedstock supply chain cost. This will reduce long-term operational risk to a biorefinery.

By 2050, the United States aims to decarbonize the aviation sector and biofuels made from renewable biomass and waste resources will be a key contributor. To maintain and optimize long term economical performance, the biofuel industry needs to overcome the supply chain barrier caused by temporal and spatial variability of biomass yield and quality. In summary, this study highlights the importance of incorporating spatial and temporal variability of biomass yield and quality during biomass supply chain planning via robust optimization modeling with the incorporation of ten years of drought index data, a primary factor contributing to yield and quality variability. Primarily, variability considered in this study is carbohydrate content, yield since they are directly correlated with weather index. The proposed modeling framework can be utilized by the industry, as well as the research community at large, for strategic analysis of the biomass feedstock supply chain (Fig. 2).

## Supplementary Information

Supplementary Information.

## Data Availability

Supplementary information containing the input data of feedstock logistics analysis is provided with this paper. Source code is available for this paper at https://github.com/Roni-ms/MFSCO/tree/Multiperiod_MFSCO.
