# Supplementary material for: Importance of incorporating spatial and temporal variability of biomass yield and quality in bioenergy supply chain
Source: Sci Rep. 2023 Apr 26;13:6813. doi: 10.1038/s41598-023-28671-4 (PMC10133216; doi:10.1038/s41598-023-28671-4)
Supplement: Supplementary file 1 — Supplementary Information. [file 41598_2023_28671_MOESM1_ESM.docx]

# INPUT DATA OF FEEDSTOCK LOGISTICS

### Harvest and collection:

The harvest and collection design in this study utilizes corn stover biomass. Corn stover harvest is assumed to be available via two different harvesting methods, three-pass (conventional) harvesting and two-pass harvesting (advanced). Conventional three-pass harvesting has the advantage of high yield, but the disadvantage of low quality with respect to carbohydrates (lower) and ash (higher). Two-pass harvesting allows better quality but decreases the harvesting yield. Conventional three-pass systems involve cutting the feedstock, collecting the material into a windrow, and then baling the windrowed material (Figure S-1). The two-pass collection method eliminates the windrowing step and thereby reduces the potential for soil contamination [[1](#_ENREF_1), [2](#_ENREF_2)]. In this method, the combine drops the material other than grain (MOG) into a loose windrow, which is followed by a separate baler. The two-pass method assumed here is that utilized by POET-DSM’s Advanced Biofuels’ Project Liberty. Drawbacks to this method are reduced collection efficiency (due to a higher cut height) and field “striping”[[2](#_ENREF_2)] as a result of uneven residue removal. Two-pass collection does not increase the required throughput to the combine or hinder its operation but requires some minor operational modifications. Combine operation is altered by disengaging the straw choppers at the rear of the combine to allow the MOG to drop behind the combine into a loose windrow. The combine is modified by adding “stalk-stompers” or by mounting rollers under the header to bend the lower stalk over in the rows on which the MOG will be dropped. These devices are commonly (but not universally) used under the wheel-track rows to reduce the risk of tire punctures. They are optional equipment but are relatively inexpensive.

*Figure S-1: Harvest and collection operations for corn Stover. Prior to baling it is assumed some amount of field drying occurs to reach 30% moisture for corn stover; CH = carbohydrate content, MC = moisture content, and PS=particle size.*

Table S-1 summarizes the harvest and collection design assumptions for the economic analysis in this paper. The assumed yield, capacity, and efficiency of harvest and collection equipment, moisture content, and ash content have been estimated based on published data [[3-7](#_ENREF_3)], data from field trials [[8-10](#_ENREF_8)], and data taken from the INL Bioenergy Feedstock Library [[11](#_ENREF_11)].

*Table S-1:* *Harvest and collection design assumptions for three-pass corn stover, two-pass corn stover and switchgrass.*

| Component | Corn stover | |
| --- | --- | --- |
|  | **Three-Pass Harvest** | **Two-Pass Harvest** |
| Harvest time |  |  |
| Operational hours | 6 weeks/year, 6 days/week, 14 hour/days | 6 weeks/year, 6 days/week, 14 hour/days |
| Stalk chopping windrower |  | |
| Capacity | 46538.89 m^2^/hour | NA |
| Efficiency | 80% | NA |
| Bale wagon/stacker |  |  |
| Capacity | 12 bales/load | 12 bales/load |
| Baler^a^ |  |  |
| Capacity | 50 bales/hour | 25 bales/hour |
|  |  |  |
| Harvest yield | 0.269 kg/ m^2^ | 0.157 kg/ m^2^ |

*Table S-2: Harvested biomass and compositions for as-harvested biomass*[[11-13](#_ENREF_11)]

| Harvest Method | Three-Pass | Two-Pass |
| --- | --- | --- |
| Moisture Content ( % wb) | 30.0% | 30.0% |
| Total Carbohydrates (% db) | **55.2%** | **57.6%** |
| Composition (all % db) |  |  |
| *total anhydro-C6* | 35.5% | 37.0% |
| *total anhydro-C5* | 20.5% | 21.4% |
| *lignin* | 14.8% | 14.8% |
| *ash* | **11.0%** | **7.0%** |
| *other* | 18.3% | 19.8% |

### Storage

Storage involves stockpiling material to provide an adequate lead time for downstream processes and accumulating material quantities for economical transportation. Biomass storage systems considered the composition of biomass and the moisture and dry-matter loss of stored material. Average distribution of dry-matter losses among corn stover components and storage design assumptions for this analysis are the listed in Table S2. Additional storage assumptions are shown in Table S-3.

*Table S-3:Average distribution of dry-matter losses among corn stover components observed in 3-month storage tests at the INL storage simulators with initial moisture contents ranging from 20 to 52% [*[*14*](#_ENREF_14)*].*

| Component | Fraction of Dry Matter Lost (%) |
| --- | --- |
| Total C6 | 18.46% |
| Total C5 | 28.93% |
| Lignin | 6.45% |
| Ash | 0.00% |
| Protein | 3.00% |
| Extractives | 29.99% |
| Acetate | 13.18% |
| Sum | 100.00% |

*Table S-4****.*** *Field storage design assumptions for storage cost estimation*

| Component | Corn stover |
| --- | --- |
| Storage moisture content | 30% |
| Storage dry matter loss | 12%/year |
| Storage moisture loss | 5%/year |
| Stack configuration | 4 x 4 tarped |

### Preprocessing

The goal of preprocessing is to increase feedstock quality and uniformity to meet reactor in-feed requirements and decrease transportation and handling costs further along the supply chain. Preprocessing operations for three-pass corn stover and two-pass corn stover are shown in Figure S-2. In this study, preprocessing incorporates two-stage size reduction with fractional milling [[15](#_ENREF_15), [16](#_ENREF_16)] and high‑moisture pelletization [[17](#_ENREF_17), [18](#_ENREF_18)]. Fractional milling introduces a separations step between the primary and secondary size reduction operations (the bale processor and hammer mill, respectively) to remove the material that already meets size specification. Material that is removed does not undergo further size reduction, thereby reducing the amount of material that flows through the hammermill and reducing the required capacity of the hammer mill [[16](#_ENREF_16)]. Preprocessing design assumptions for this analysis are listed in the in Table S5. Input parameters (such as throughput and energy consumption) are estimated based on published data from the Biomass Feedstock National User Facility[[19](#_ENREF_19)].

*Figure S-2: Preprocessing configurations for corn stover. Preprocessing also shows the change in feedstock composition, format, and particle size; CH = carbohydrate content, MC = moisture content, PS = particle size.*

*Table S-5: Summary of preprocessing assumptions. The benefit of fractional milling is included by adjusting the throughput and energy consumption of 2nd stage grinding.*

| **Component** | **Three-Pass & Two-Pass Corn Stover** |
| --- | --- |
| **Location of operation** | Depot |
| **Stage 1 size reduction** |  |
| Grinder type | Bale processor |
| Screen Size (inch) | NA |
| Energy (kWh/dry Mg) | 8.82 |
| Throughput (dry Mg/hour/machine) | 9.07 |
| Operating conditions (moisture %) | 25.0% |
| **Separations** |  |
| Screen type | Disc Screen |
| Energy (kWh/dry Mg) | Minimal electricity |
| Throughput (dry Mg/hour/machine) | 9.07 |
| Operating conditions (moisture %) | 24.0% |
| Bypass | 30% |
| **Stage 2 Grinder** |  |
| Comminution method | Hammer mill |
| Screen Size (cm) | 0.635 |
| Energy (kWh/dry Mg) | 55.13 (38.59^a^) |
| Throughput (dry Mg/hour/machine) | 1.42 (2.03^a^) |
| Operating conditions (moisture %) | 22.0% |
| **Densifier** |  |
| Densifier type | Pellet mill |
| Energy (kWh/dry Mg) | 37.25 |
| Throughput (dry Mg/hour/machine) | 3.29 |
| Operating conditions (moisture %) | 17.0% |
| Pellet density (kg/m^3^) | 631.45 |
| Pellet durability | 98.70% |
| **Cooler** |  |
| Moisture removed | 1.70% |
| Energy (kWh/dry Mg) | 3.33 |
| Throughput (dry Mg/hour/machine) | 4.54 |
| a: The effective energy consumption is reduced because only 70% of the material is processed in Stage 2 due to fractional milling. The effective throughput is improved because only 70% of the material is processed in Stage 2 due to fractional milling | |

### Depot construction cost for different depot sizes

Construction and infrastructure costs for depots were estimated as follows. For a fixed depot size, the total installed capital investment cost per ton was estimated for the preprocessing, storage and handling operations in the depot. The installed capital cost included all preprocessing, handling and storage equipment; the estimate included instrumentation and control, piping and electrical installation, yard improvement, engineering and supervision, contractor fees, construction and contingency. To estimate the capital layout for construction and infrastructure for individual preprocessing equipment similar to the equipment in this design, an installation factor value of 1.49 was applied, estimated based on [[20](#_ENREF_20)]). Land cost was calculated assuming 160 acres per distributed depot (including onsite bale storage) at a cost of $500/acre and was added to the capital cost to determine the loan amount. The required acreage for a 657,575 dry Mg/year depot (including onsite bale storage) was estimated at 226 acres. The total cost was amortized over 30 years, assuming a 20% down payment and an 8% interest rate, and divided by the number of delivered tons to give the per ton cost of depot construction and infrastructure, which totaled $2.53/dry Mg for a depot scaled to 657,575 dry Mg/year. The above steps were repeated for depot scales ranging from 22,675-634,900 dry Mg/year, and the results are shown in Figure S-3.

*Figure S-3: Estimated depot construction costs as a function of depot scale.*

### Transportation and Handling

Transportation cost is estimated based on biomass physical characteristics and equipment used during transportation (Table S6). The total transportation costs for bales and pellets (including loading and unloading) are shown as a function of distance utilized in regression model is shown in Table S-7.

*Table S-6. Transportation and handling design assumptions in this study*

| **Component** | **Three-pass corn Stover** | **Two-pass corn Stover** |
| --- | --- | --- |
| Format | Bale | Bale |
| Density | 192.22 kg/m^3^ | 192.22 kg/m^3^ |
| Moisture content | 25% | 25% |
| Format | Bulk pellets | Bulk pellets |
| Density | 631.45 kg/m^3^ | 631.45 kg/m^3^ |
| Moisture content | 11.53% | 11.53% |
| Speed | 80.5 km/hour | 80.5 km/hour |
| Type | Day cab | Day cab |
| Type | 53-ft flatbed with ALSS | 53-ft flatbed with ALSS |
| Volume | 102 m^3^ | 102 m^3^ |
| Type | Trailer "Live Floor" 48 feet 2-axle | Trailer "Live Floor" 48 feet 2-axle |
| Volume | 102 m^3^ | 102 m^3^ |
| **Bale Loader** |  |  |
| Capacity | 108.84 Mg/hour | 108.84 Mg/hour |

*Table S-7. Total transportation costs for biomass bales and blendstock pellets.*

| Distance km) | Bale Transportation Costs | Pelleted Blendstock Transportation Costs |
| --- | --- | --- |
|  | Cost ($/dry Mg) | Corn Stover ($/dry Mg) |
| 16.1 | $5.04 | $1.83 |
| 32.2 | $6.31 | $2.75 |
| 48.3 | $7.53 | $3.67 |
| 64.4 | $8.82 | $4.59 |
| 80.5 | $10.06 | $5.46 |
| 96.6 | $11.29 | $6.38 |
| 112.7 | $12.57 | $7.31 |
| 128.8 | $13.80 | $8.22 |
| 144.9 | $15.09 | $9.11 |
| 161 | $16.32 | $10.02 |
| 193.2 | $18.84 | $11.86 |
| 225.4 | $21.36 | $13.66 |
| 257.6 | $23.88 | $15.51 |
| 289.8 | $26.41 | $17.30 |
| 322 | $28.92 | $19.16 |
| 354.2 | $31.38 | $20.95 |
| 386.4 | $33.89 | $22.80 |

*Table S-8. Assumed energy prices and interest rates used to model herbaceous feedstock logistics costs for the case study.*

| **Component** | **Assumptions** |
| --- | --- |
| **Interest Rate** | 8%^a^ |
| **Electricity Price** | $0.0671/kWh^b^ |
| **Natural Gas Price** | $5.1/KJ^b^ |
| **Off-Road Diesel Price** | $0.86/L^b^ |
| a. See Jones et al. [[21](#_ENREF_21)]  b. See EIA [[22-24](#_ENREF_22)] | |

### Dockage

Dockage is a cost penalty that is incurred if the feedstock does not meet the conversion in-feed specifications. If the blended feedstock does not meet the conversion in-feed ash specification (≤ 5%), an ash dockage is incurred that is equivalent to the additional ash disposal cost incurred by the biorefinery. Ask dockage captures the ash disposal costs and are assumed to be $41.48/dry Mg of ash. If the moisture content of the blended feedstock is lower than the moisture specification of 20%, a moisture dockage is applied. Delivering the feedstock blend lower than 20% moisture would require additional make-up water. This value was calculated from the assumed make-up water cost of $0.34/dry Mg of water used by Davis et al. (2013)[[25](#_ENREF_25)].A carbohydrate dockage is applied if the blended feedstock does not meet the conversion in-feed carbohydrate specification. A carbohydrate dockage is calculated by quantifying loss of yield caused less carbohydrate material and is estimated as $323.04/dry Mg based on fuel yield 48.51 GGE/dry Mg and $3.87/GGE [[26](#_ENREF_26)].

### Delivered feedstock composition assumptions

The analysis in this study assumes herbaceous feedstock, with biochemical conversion in-feed feedstock compositional specifications presented in Table S-8The shaded rows in Table S-8 show the compositional specifications for the feedstock, namely, 59% carbohydrates, ≤ 5 % ash, and 20% moisture. An additional specification is ¼” mean particle size at the pretreatment reactor throat

**Table S-8.** Delivered feedstock composition assumptions for dilute-acid pretreatment and enzymatic hydrolysis to sugars followed by biological conversion of sugars to hydrocarbons pathway[[26](#_ENREF_26)]

| **Component** | **Composition  (dry wt. %)** |
| --- | --- |
| **Glucan** | 35.05 |
| **Xylan** | 19.53 |
| **Lignin** | 15.76 |
| **Ash** | 4.93 |
| **Acetate** | 1.81 |
| **Protein** | 3.10 |
| **Extractives** | 14.65 |
| **Arabinan** | 2.38 |
| **Galactan** | 1.43 |
| **Mannan** | 0.60 |
| **Sucrose** | 0.77 |
| ***Total structural carbohydrate*** | ***58.99*** |
| ***Total structural carbohydrate + sucrose*** | ***59.76*** |
| ***Moisture (bulk wt.%)*** | ***20.0*** |

## **References**

1. Shinners, K.J., R.G. Bennett, and D.S. Hoffman, *Single and two-pass corn grain and stover harvesting.* Transactions of the ASABE, 2012. 55(2): p. 341-350.

2. Birrell, S.J., D.L. Karlen, and A. Wirt, *Development of sustainable corn stover harvest strategies for cellulosic ethanol production.* BioEnergy Research, 2014. 7(2): p. 509-516.

3. Anderson, E.K., et al., *Nitrogen fertility and harvest management of switchgrass for sustainable bioenergy feedstock production in Illinois.* Industrial Crops and Products, 2013. 48: p. 19-27.

4. Lindsey, K., et al., *Monitoring switchgrass composition to optimize harvesting periods for bioenergy and value-added products.* Biomass and Bioenergy, 2013. 56: p. 29-37.

5. Bonner, I.J., et al., *Impact of harvest equipment on ash variability of baled corn stover biomass for bioenergy.* BioEnergy Research, 2014. 7(3): p. 845-855.

6. DOE, *2016 BILLION-TON REPORT Advancing Domestic Resources for a Thriving Bioeconomy*. 2016, OAK RIDGE NATIONAL LABORATORY.

7. Owens, V.N., D.L. Karlen, and J. Lacey, *Regional Feedstock Partnership Report: Enabling the Billion-Ton Vision.*, U.S.D.o.E.a.I.N. Laboratory, Editor. 2016.

8. Smith, W. and I. Bonner, *Demonstrate through integrated model analysis using field and PDU-scale data from dry corn stover a total feedstock logistics cost of $35.00 per dry ton (excluding grower payment, in 2007$) ,FY12 Q4 Milestone Report WBS#1.2.1.1*. 2012, Idaho National Laboratory: Idaho, USA.

9. Smith, W. and I. Bonner, *Evaluation of two multi-pass stover harvest systems and their impacts on the interactions between yield and ash on the delivered feedstock price*. 2014, Idaho National Laboratory: Idaho

10. Brue, J.D., et al. *Understanding management practices for biomass harvest equipment for commercial scale operation*. in *2015 ASABE Annual International Meeting*. 2015. American Society of Agricultural and Biological Engineers.

11. INL. *Bioenergy Feedstock Library*. 2016 [cited 2016 09/07/2016]; Available from: <https://bioenergylibrary.inl.gov/Home/Home.aspx>.

12. David, K. and A.J. Ragauskas, *Switchgrass as an energy crop for biofuel production: A review of its ligno-cellulosic chemical properties.* Energy & Environmental Science, 2010. 3(9): p. 1182-1190.

13. Liu, L., et al., *Variability of biomass chemical composition and rapid analysis using FT-NIR techniques.* Carbohydrate Polymers, 2010. 81(4): p. 820-829.

14. Wendt, L., et al., *Quantify structural sugar loss in storage relative to initial moisture content and oxygen availability* 2013, Idaho National Laboratory Idaho ,USA.

15. Yancey, N. and T. JayaShankar, *Size Reduction, Drying and Densification of High Moisture Biomass*. 2015, Idaho National Laboratory: Idaho Fall, Idaho, USA.

16. Yancey, N., C.T. Wright, and T.L. Westover, *Optimizing hammer mill performance through screen selection and hammer design.* Biofuels, 2013. 4(1): p. 85-94.

17. Tumuluru, J.S., *Effect of process variables on the density and durability of the pellets made from high moisture corn stover.* Biosystems Engineering, 2014. 119: p. 44-57.

18. Tumuluru, J.S., *High moisture corn stover pelleting in a flat die pellet mill fitted with a 6 mm die: physical properties and specific energy consumption.* Energy Science & Engineering, 2015. 3(4): p. 327-341.

19. INL. *Biomass Feedstock National User Facility*. 2017 [cited 2017 5/4/2017]; Available from: <https://bfnuf.inl.gov/SitePages/BFNUF%20Home.aspx>.

20. Peters, M.S., C.D. Timmurhaus, and R.E. West, *Plant design and economics for chemical engineers*. Vol. 4. 1968, New York, NY: McGraw-Hill.

21. Jones, S., et al., *Process design and economics for the conversion of lignocellulosic biomass to hydrocarbon fuels*. 2013.

22. EIA. *Electric Power Monthly: Table 5.6.A Average Retail Price of Electricity to Ultimate Customers by End-Use Sector*. 2016 [cited 2016; Available from: <https://www.eia.gov/electricity/monthly/>.

23. EIA. *Gasoline and Diesel Fuel Update*. 2016 [cited 2016 8/8/2016]; Available from: <https://www.eia.gov/petroleum/gasdiesel/>.

24. EIA. *Natural Gas: Henry Hub Natural Gas Spot Price-Annual History*. 2016 [cited 2016 8/8/2016]; Available from: <https://www.eia.gov/dnav/ng/hist/rngwhhdm.htm>.

25. Davis, R., et al., *Process Design and Economics for the Conversion of Lignocellulosic Biomass to Hydrocarbons: Dilute-Acid and Enzymatic Deconstruction of Biomass to Sugars and Biological Conversion of Sugars to Hydrocarbons*. 2013, National Renewable Energy Laboratory, Idaho National Laboratory, Harris Group Inc.: Golden, CO, USA.

26. Davis, R.E., et al., *Process design and economics for the conversion of lignocellulosic biomass to hydrocarbon fuels and coproducts: 2018 Biochemical design case update; Biochemical deconstruction and conversion of biomass to fuels and products via integrated biorefinery pathways*. 2018, National Renewable Energy Lab.(NREL), Golden, CO (United States).
